# Hedgehogs and *Angiostrongylus cantonensis*: Uncovering the Role of *Atelerix albiventris* in the Parasite Life Cycle

**DOI:** 10.1111/1749-4877.13004

**Published:** 2025-05-21

**Authors:** Anna Šipková, Kristýna Javorská, Lucia Anettová, Divakaran Pandian, Petr Cibulka, Jana Kačmaříková, Ladislav Novotný, David Modrý

**Affiliations:** ^1^ Department of Botany and Zoology, Faculty of Science Masaryk University Brno Czech Republic; ^2^ University of Veterinary Sciences Brno Brno Czech Republic; ^3^ Institute of Parasitology Biology Center of Czech Academy of Sciences Ceske Budejovice Czech Republic; ^4^ Department of Veterinary Sciences, Faculty of Agrobiology, Food and Natural Resources/CINeZ Czech University of Life Sciences Prague Praha Czech Republic; ^5^ Department of Comparative Pathobiology, College of Veterinary Medicine Purdue University West Lafayette Indiana USA

**Keywords:** *Angiostrongylus cantonensis*, experimental infection, hedgehogs, Mediterranean region

## Abstract

The ongoing invasion of the zoonotic parasite *Angiostrongylus cantonensis* threatens humans, other mammals, and birds. In Mallorca, neurological disorders associated with this parasite are increasingly reported in free‐living Algerian hedgehogs, *Atelerix algirus*, raising public health concerns. Experimental infections in the related species *Atelerix albiventris* were conducted to investigate the role of hedgehogs in the life cycle of the parasite. Captive‐born hedgehogs were orally inoculated with 200 and 2000 third‐stage larvae and monitored for 50 days by behavioral observations, blood tests (hematology and biochemistry), histopathology, and qPCR analysis. Baermann's larvoscopy was used to examine feces starting from day 39 post‐infection (DPI). Neuropathological symptoms were only observed in the high‐dose group, with larvae detected in brain tissue at 5, 15, 23, and 44 DPI. In contrast, no clinical symptoms or larvae in tissues were found in the low‐dose group. Parasite DNA was present in the organs of both groups, but neurological migration occurred exclusively in the high‐dose group. Blood tests showed no specific patterns indicating infection. Hedgehogs are aberrant hosts of *A. cantonensis*, showing severe neuropathological symptoms at high infection levels (2000 third‐stage larvae), while lower doses remain asymptomatic. The parasite cannot complete its life cycle in *A. albiventris* hedgehogs, which do not contribute to its ecological circulation. However, hedgehogs can serve as sentinels for the spread of *A. cantonensis* in newly invaded regions like Mallorca. Monitoring neurological disease in wildlife could provide valuable insights into the potential spread of this zoonotic parasite in temperate latitudes.

## Graphical abstract

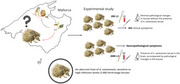

## 1 Introduction

Biological invasions pose a significant threat to global biodiversity. The invasion of non‐native animal species into a new ecosystem is often accompanied by an invasion of symbiotic organisms, including parasitic helminths (Dunn 2009; Lodge et al. 2000; Prenter et al. 2004). *Angiostrongylus cantonensis* (rat lungworm) is a zoonotic metastrongyloid nematode (Metastrongyloidea: Angiostrongylidae) first described in Canton, China (Chen 1935). Rats of the genus *Rattus*, in particular *Rattus norvegicus* (Berkenhout, 1769) and *Rattus rattus* (Linnaeus, 1758), are typical definitive hosts, while a large number of gastropods act as intermediate hosts (Cowie et al. 2023). The global spread of intermediate and definitive hosts facilitated the worldwide spread of *A. cantonensis* in the tropics and subtropics (Lv et al. 2011; Prociv and Carlisle 2001; Rael et al. 2018; Thiengo et al. 2010).

In rats, the third‐stage larvae (L3) exhibit obligate neurotropism, that is, they migrate from the gastrointestinal tract through the central nervous system (CNS) to the right ventricle and pulmonary arteries, where reproduction takes place. The first larval stage (L1) hatches from the egg, migrates up the trachea, and is subsequently coughed up, swallowed, and passed in the feces, where it encounters intermediate hosts and develop into L3 (Bhaibulaya 1975; Cowie 2013).

Larval neurotropism poses a significant threat to aberrant hosts infected after ingestion of L3 from gastropods (intermediate hosts) or poikilothermic vertebrates (paratenic hosts) or invertebrates (transport hosts) (Ash 1968; Anettová et al. 2022; Turck et al. 2022). In humans as well as in a number of homeothermic vertebrates other than rats, the larvae that migrate into the CNS do not reach maturity but cause neurological disorders of varying intensity (Cowie 2017). This condition is known as neuroangiostrongyliasis (NA) and is a common cause of eosinophilic meningitis in endemic areas (Alicata 1991; Cowie et al. 2022; Rosen et al. 1962); the larvae can also cause severe ocular manifestation if they enter the eye (Diao et al. 2011).

Notable cases of NA in animals have been reported in dogs and horses (Costa et al. 2000; Lunn et al. 2012; Wright et al. 1991), Australian wildlife (e.g., fruit bats, possums, and macropods), birds such as tawny frogmouths, *Podargus stigoides* (Lantham, 1801), armadillos, and a range of zoo animals, including non‐human primates, the African pygmy falcon, *Polihierax semitorquatus* (A. Smith, 1836), and cockatoo species (Burns et al. 2014; Carlisle et al. 1998; Dalton et al. 2017; Duffy et al. 2004; Emerson et al. 2013; Gardiner et al. 1990; Rizor et al. 2022; Spratt 2015).

Infected animals exhibit weakness, anorexia, impaired locomotion, seizures, and paralysis, often leading to death (Carlisle et al. 1998; Kim et al. 2002; Patial et al. 2022). The pathological effects result from mechanical damage to the CNS caused by larval migration (Chotmongkol and Sawanyawisuth 2002; Pan et al. 2000); and inflammation triggered by larval antigens (Ishida and Yoshimura 1990; Perez et al. 1989; Summers et al. 1995). In some cases of NA, mature adult worms have been found in the brain or lungs of humans and other non‐murid mammals (Delgado‐Serra et al. 2022; Edwards et al. 2020; Kottwitz et al. 2014; Paredes‐Esquivel et al. 2019; Pirisi et al. 1995; Shamsi et al. 2019; Tuan et al. 2017).

In 2018, Algerian hedgehogs, *Atelerix algirus* (Lereboullet, 1842), with neurological disorders were found on the Mediterranean island of Mallorca, and the nematodes observed in the brain were subsequently identified morphologically and molecularly as *A. cantonensis* (Paredes‐Esquivel et al. 2019). Further observation revealed additional cases of neurological disorders in hedgehogs associated with *A. cantonensis* infection (Delgado‐Serra et al. 2022; Arango‐Colonna et al. 2023). Clinically affected hedgehogs showed ataxia (circling movements), atonia, astasia, paresis of the thoracic limbs, asthenia, lateral recumbency, and a deterioration in behavior. Nematodes in the subadult and adult stages were found exclusively in the brain; none was found in the lungs or heart.

The emergence of NA in Algerian hedgehogs in Mallorca not only revealed the first focus of infection in the Mediterranean region, but also raised important questions about the potential impact on hedgehog populations and the role of these insectivores in the further spread of the parasite. Our objective was to address the significant knowledge gap regarding *A. cantonensis* infection in insectivores by investigating the development and associated pathologies in experimentally infected African pygmy hedgehogs, *Atelerix albiventris* (Wagner 1841), and to clarify the role of the hedgehog in the life cycle of the parasite, including the impact of the infective dose on disease progression.

## 2 Materials and Methods

### 2.1 Experimental Strain of *A. cantonensis* and Experimental Animals

The isolate of *A. cantonensis* originates from Tenerife, Canary Island; the life cycle is maintained in the laboratory between laboratory rats (*R. norvegicus*, Wistar strain) and African giant snails, *Lissachatina fulica* (Férussac, 1821) (Pulmonata: Achatinidae). The identity of the isolate was confirmed based on the morphology of adult nematodes recovered from infected rats and by cox1 sequencing, where the haplotype was identified as part of the *A. cantonensis* clade 2 (Červená et al. 2019).

Experimental captive‐born African pygmy hedgehogs *A. albiventris* (9 adult males and 4 adult females) were provided by a private breeder. The experimental animals were acclimatized for 14 days before the start of the experiment. The animals were examined coproscopically with negative results using Sheather's sugar flotation and Baermann's larvoscopy.

Experimental hedgehogs were kept individually in an isolated room in cages (floor area ∼4150 cm^2^) at a constant temperature of 25°C. The animals were checked daily and provided with food (a combination of mealworms and kitten granules) and water *ad libitum*. Wood pellets were used as bedding and the cages were equipped with shelters and carousels.

Two laboratory rats (*R. norvegicus*, Wistar strain) were purchased as positive controls from the Laboratory Animal Breeding and Experimental Facility of Masaryk University, Brno, Czech Republic. They were housed in plastic cages (floor area ∼2088 cm^2^) with wooden pellets, equipped with shelters, and received food and water *ad libitum*. All animal experimentation requirements were rigorously adhered to in accordance with established guidelines and protocols. Animal handling and experimental procedures were conducted in full compliance with ethical standards and under the authorization of the Ministry of Education, Youth and Sports (No. MSMT‐17988/2022‐4).

### 2.2 Experimental Infection

The third‐stage larvae of *A. cantonensis* were isolated from the tissue of experimentally infected *L. fulica* by artificial digestion. The molluscs were reared in an invertebrate breeding facility at the University of Veterinary Sciences Brno. After sacrificing by decapitation, the foot muscle was digested in digestive fluid (i.e., 0.3 g pepsin in 100 mL 0.7% HCl) on a magnetic stirrer at 600 rpm at 37°C for 2 h as described by Modrý et al. (2021). The digested tissue was transferred through a sieve into 50 mL Falcon tubes and centrifuged (1 min, 1500 *g*). The sediment was examined in a Petri dish under a light microscope (40× magnification), where live, motile larvae were individually collected into 2 mL tubes using an automatic pipette to prepare experimental inocula. Hedgehogs in experimental group A were inoculated with an inoculum of 200 larvae per individual (A1–A6) via an esophageal tube under isoflurane inhalation anesthesia, while hedgehogs in group B received 2000 larvae per individual (B1–B5). Prior to anesthesia, the animals were administered midazolam (1 mg/kg, MSD Animal Health) intramuscularly to induce muscle relaxation and facilitate the application of the inhalation mask. Flumazenil (0.05 mg/kg, MSD Animal Health) was administered intramuscularly to neutralize the effect of midazolam after the procedure. The experimental infections were performed in two consecutive experiments: Group A was infected first, followed by Group B after completion of the first experiment. The experimental conditions, including methods and materials, were the same in both groups.

Two hedgehogs served as uninfected negative controls receiving basic saline solution via an esophageal tube (NC1 and NC2). Two laboratory rats, which served as positive controls, were inoculated in the same way as the hedgehogs, though with a lover dose (40 L3) to prevent unwanted clinical disease or death.

### 2.3 Blood Collection and Examination

**FIGURE 1:**
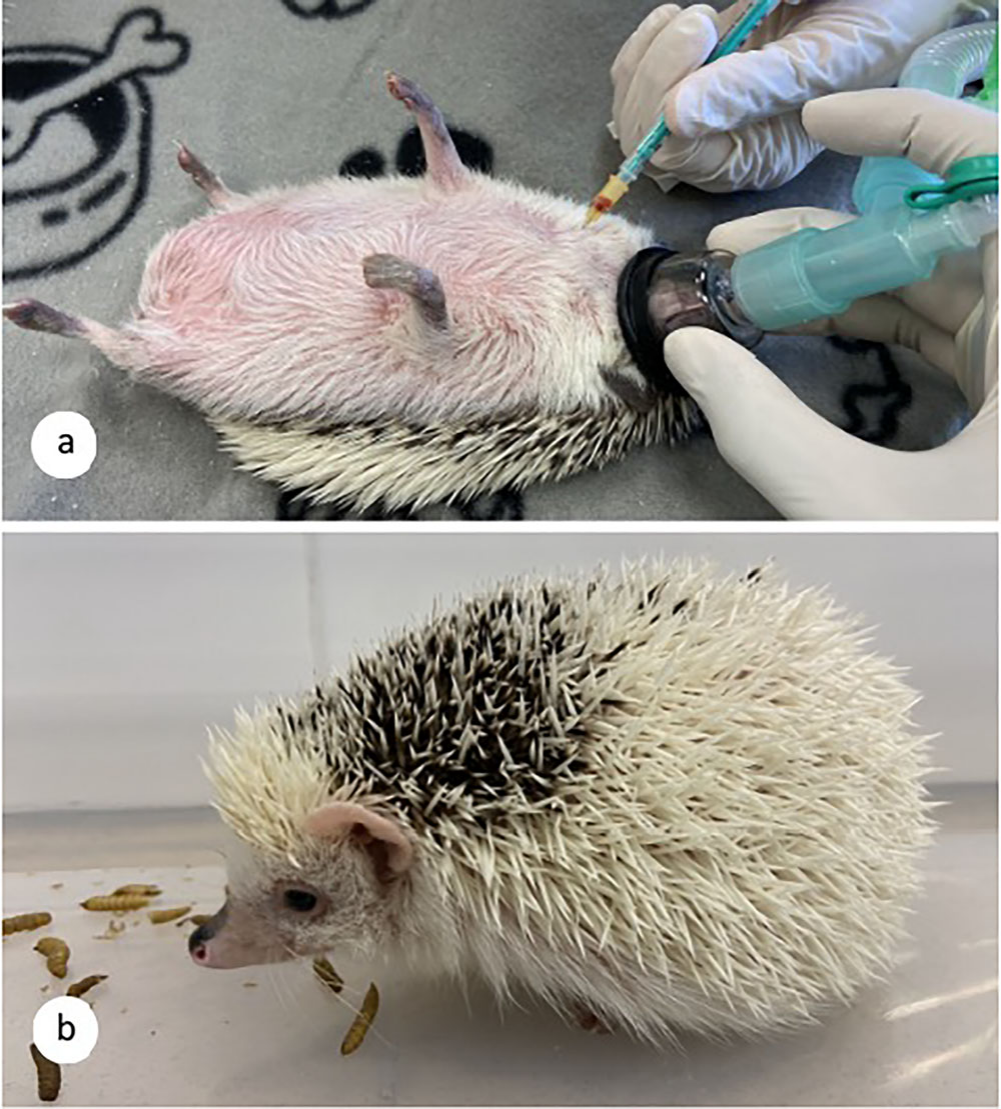
Experimental hedgehogs. (a) Blood collection from the *vena cava cranialis* under isoflurane inhalation anesthesia; (b) behavior changes associated with clinical symptoms were observed during feeding on insect larvae.

Approximately 1 mL of blood was taken from the *vena cava cranialis* of all hedgehogs at three time intervals, always under inhalation anesthesia (Figure 1a). The first sample was taken shortly before inoculation (DPI 0), the second at 15 DPI, and the last sample on the day of euthanasia (Table 1). The biochemical and hematological parameters (Supporting Information S1 and S2) were measured immediately after blood collection using Idexx Catalyst One and Idexx ProCyte Dx (IDEXX Laboratories, USA).

**TABLE 1:** Summary of clinical and histopathological examination including weight differences and days of euthanasia.

Hedgehog	Inoculum	Euthanasia [DPI]	Weight difference [g]	Clinical symptoms	Histopathology
A1 (M)	200 L3	30	44*	None	None
A3 (M)	200 L3	30	45*	None	None
A5 (F)	200 L3	30	43*	None	None
A2 (M)	200 L3	50	36*	None	None
A4 (M)	200 L3	50	44*	None	‐ Hepatitis, necrotizing to pyogranulomatous, multifocal
A6 (F)	200 L3	50	18*	None	None
B1 (M)	2000 L3	5	NA	‐ 4 DPI: Cachexia, tendency to curl up when touched, blepharoedema, tremor, ataxia (circling movements), anorexia ‐ 5 DPI: Lethargy, constant tremor	‐ Liver lipidosis, mild to moderate ‐ Extramedullary hematopoiesis in spleen ‐ Dilation of Bowman spaces ‐ Multiple granulomas with larvae within the lumen of lymphatic vessels in the submucosa of pylorus ‐ One larva in white matter of the brain
B2 (M)	2000 L3	23	10	‐ 22 DPI: Ataxia, hypotonic forelimbs, persistent tremor, inability to curl up, hyperkinetic movements, dyspnoea, hissing, anorexia ‐ 23 DPI: Permanent tremor, pronounced dyspnoea, hissing, lowered head, high sensitivity to touch, blepharoedema	‐ Liver lipidosis, mild ‐ Extramedullary hematopoiesis in spleen ‐ Meningitis, lymphohistiocytic to pyogranulomatous, moderate to marked ‐ Occasional subadults/young adults in the brain chambers (up to 5 larvae)
B3 (M)	2000 L3	23	−8	‐ 23 DPI: Lateral recumbency, dyspnoea, rapid deterioration of health in 4 h	‐ Focal erosive glossitis ‐ Meningitis, lymphohistiocytic to pyogranulomatous, moderate to marked ‐ Multiple subadults/young adults in the brain chambers (up to 10 larvae)
B4 (M)	2000 L3	15	6	‐ 15 DPI: Significant blepharoedema, ataxia (circling movements and lateral instability), dyspnoea, sneezing, anorexia	‐ Occasional crypt abscessi in rectum ‐ Meningitis, lymphohistiocytic to pyogranulomatous, moderate to marked ‐ Occasional subadults/young adults in the brain chambers (up to 5 larvae)
B5 (M)	2000 L3	44	−5	‐ 33 DPI: Ataxia (lateral instability), the symptoms subsided in the following days; from ‐ 37 DPI to 44 DPI: Ataxia (rotation to the left side), blepharoedema	‐ Hepatitis, necrotizing to pyogranulomatous, multifocal ‐ Meningitis, lymphohistiocytic to pyogranulomatous, moderate to marked ‐ Occasional subadults/young adults in brain chambers (up to 5 larvae)

An asterisk indicates statistically significant weight changes.*p < 0.05.

Abbreviations: F, female; M, male; NA, missing measurement.

### 2.4 Clinical Observations

The animals were checked daily in the late afternoon for their food and water intake. Every second day, their mobility was assessed in a large plastic box in which they could move freely in order to observe any behavioral deviations. For 5 min, the animals' ability to walk, respond to stimuli (mealworms), and curl up into a ball was assessed (Figure 1b). Eyes and nostrils were examined for discharge. From the 39 DPI, feces were examined daily for the presence of L1 using Baermann's larvoscopy.

An extended clinical assessment and weighing were performed under anesthesia at DPI 0, DPI 15, and on the day of euthanasia (DPI 5–50). This included auscultatory examination of the heart and lungs, palpation of the abdominal cavity (including liver and kidneys), examination of the oral cavity (teeth and color of mucous membranes), anus, genital opening, paws, and claws. If changes in the animal's clinical condition were detected, the frequency of examinations was increased to three times a day. The animals were euthanized to avoid unnecessary suffering when a deterioration in condition was observed, characterized by lack of food and water intake or inability to walk.

### 2.5 Necropsy, Cerebrospinal Fluid Collection, and Histopathology

Hedgehogs were euthanized under general isoflurane inhalation anesthesia by intracardiac application of T‐61 euthanasia solution (0.3 mL/kg; embutramide, mebezonium iodide, tetracaine hydrochloride injection solution; MSD Animal Health). Immediately after euthanasia, the skin on the dorsal neck was removed, followed by collection of cerebrospinal fluid (CSF), which was frozen (−20°C) for qPCR analysis. The thoracic, abdominal, and oral cavities were opened and visually examined for abnormalities. The thoroughly examined organs in the cavities were removed together with the brain and approx. 0.5 × 0.5 cm pieces of tissue samples of each organ were collected in 2 mL tubes and frozen (−20°C) for subsequent qPCR analysis. The remaining parts of the organs were fixed in alcohol formalin glacial acetic acid (AFA) for histopathological examination (Table 2). The histological slides were prepared in the pathology laboratories of St. Anne's University Hospital in Brno; the fixed tissues were dehydrated with a graded series of dilutions (70% ethanol, 90% ethanol, 96% ethanol, acetone, and xylene), then embedded in paraffin, serially cross‐sliced (1.5 µm), and stained with H&E. For tissues in which qPCR confirmed the presence of larvae (extrapolated larval counts per gram of tissue ≥1.00), multiple histological sections were prepared through paraffin blocks. The slides were examined by a board certified veterinary pathologist (LN) under light microscope with camera attachment.

**TABLE 2:** Results of quantitative PCR analysis of tissues from groups A and B; highlighted cells indicate positive *A. cantonensis* DNA findings. The values in brackets correspond to the number of larvae per gram of tissue, extrapolated from the qPCR Ct values. Cells marked in red indicate tissues in which the presence of *A. cantonensis* larvae was confirmed histopathologically.

	Group A						Group B				
	30 DPI			50 DPI			5 DPI	15 DPI	23 DPI		44 DPI
**Tissue**	**A1**	**A3**	**A5**	**A2**	**A4**	**A6**	**B1**	**B4**	**B2**	**B3**	**B5**
**Heart**	N	N	N	P	P	P	N	N	P (0.001)	N	N
**Lung**	N	N	N	N	P	P	N	N	P (5.85)	N	N
**Liver**	N	N	N	N	P	N	P (0.02)	N	P (0.12)	N	N
**Spleen**	N	N	N	N	N	N	P (1)	N	P (0.07)	N	N
**Stomach 1**	N	N	N	N	P	P	P (0.03)	P (2.27)	N	N	N
**Stomach 2**	N	N	N	N	P	P	P (0.01)	N	N	N	N
**Duodenum**	P	N	N	N	N	N	P (0.03)	N	N	N	N
**Jejunum**	N	N	N	N	N	N	P (0.13)	N	N	N	N
**Ileum**	N	N	P	N	N	N	P (0.2)	N	N	N	N
**Colon**	N	N	N	N	P	N	P (0.02)	N	N	N	N
**Rectum**	N	N	N	N	P	P	P (0.12)	N	N	N	N
**Kidney**	P	N	N	N	N	N	P (0.01)	P (0.0004)	P (0.001)	N	N
**Testes/ovaries**	N	N	N	N	P	N	P (0.09)	N	N	N	N
**Gluteal muscle**	N	N	N	N	N	N	N	N	P (0.002)	N	N
**Pectoral muscle**	N	N	N	N	N	N	P (0.79)	N	P (0.07)	N	N
**Diaphragm**	N	P	N	P	P	N	P (0.003)	N	P (0.002)	N	N
**Tongue**	N	P	P	N	N	P	P (0.6)	N	N	N	N
**Brain**	P	N	P	P	P	N	P (277.7)	P (7.32)	P (3.19)	P (0.06)	P (1.53)
**CSF**	N	N	N	N	N	N	NA	P (0.0007)	P (1.86)	P (1992)	P (0.0031)

Abbreviations: N, negative; NA, unsuccessful CSF collection; P, positive; Stomach 1, cranial part; stomach 2, caudal part.

### 2.6 DNA Extraction, qPCR Analysis, and Quantitative Analysis

DNA from all sampled organs and CSF was isolated using the DNEasy Blood and Tissue (Qiagen, Germany) extraction kit, which was optimized for L3 of *A. cantonensis* when the pre‐lyse phase was extended overnight. A species‐specific qPCR assay was performed on the LightCycler 480 to detect the presence of *A. cantonensis* DNA in tissues (Sears et al. 2021). The assay was performed in a 20 µL reaction mix containing 6.2 µL of 2× Mastermix (IDT PrimeTime Gene Expression Master Mix); 0.2 µL of 10 µM probe (PrimeTime Eco Probe 5′ 6‐FAM/ZEN/3′ IBFQ, /56‐FAM/ACA TGA AAC/ZEN/ACC TCA AAT GTG CTTCGA/3IABkFQ/); 0.8 µL of 10 µM primers (forward: AAA CTG TTG CTT TCG AAG and reverse: GCG CAA ATC TGA CGT TCT TG); 6.2 µL of DNA, DNase‐, and RNase‐free water (PCR water); and 2 µL of DNA template. The cycling conditions (40 cycles) were set as follows: 95°C for 20 s, 40°C for 1 s, and 60°C for 20 s. DNA from a single L3 of *A. cantonensis* isolated by the same procedure was used as a positive control; PCR water was used as a negative control. Samples were analyzed in duplicate; Ct values not exceeding 35 were considered positive. To estimate the number of larvae in the analyzed material from group B, Ct values were converted to the estimated number of larvae in the tissue samples using a standard curve generated from serial dilutions (1×, 10×, 100×, and 1000×) of DNA extracted from a single L3. This standard curve was used for all qPCR runs using a positive control with a 100× dilution as a calibrator. Subsequently, the DNA concentration for each sample was adjusted according to the actual weight of the tissue sample used for the extraction process.

### 2.7 Statistical Analysis

Statistical analyses were performed with data sets containing hedgehog weights, blood parameters, and qPCR results. A paired *t*‐test was used to assess the significance of weight changes within groups A and B. The Kruskal–Wallis test was used to compare the weight differences between the experimental groups (A and B) and to analyze the blood parameters for significant changes related to the infection. Blood parameters were compared at three time points: on the day of infection (DPI 0), at 15 DPI, and on the day of euthanasia (DPI 5–50). Hedgehog B1 was excluded from the statistical analyses because of insufficient data on weight and blood parameters, as it was euthanized early on DPI 5. In case of a significant result of the Kruskal–Wallis test, post hoc pairwise comparisons were performed using Dunn's test with Bonferroni correction to control for multiple comparisons. Fisher's exact test was used to assess differences in the number of positive *A. cantonensis* DNA tissues from hedgehogs in group A euthanized on 30 and 50 DPI. The Cochran–Armitage trend test analyzed the trend in the number of positive *A. cantonensis* DNA in tissues from group B. Differences in the estimated number of larvae in tissues within group B were analyzed using the Kruskal–Wallis test. No statistical test was performed for the negative control group because of the small sample size (*n* = 2). Statistical significance was defined as *p* < 0.05. Adjusted *p*‐values (p.adj) were used for post hoc tests, with significance defined as p.adj < 0.05. All statistical analyses were performed with RStudio, version 2023.06.2 + 561.

## 3 Results

### 3.1 Neurological Symptoms in Highly Infected Hedgehogs

Group A (200 L3) showed no recognizable clinical signs after infection with *A. cantonensis*. The animals in this group were euthanized at 30 (A1, A3, and A5) and 50 (A2, A4, and A6) DPI. All individuals in group B (2000 L3) showed neurological symptoms with varying degrees and rates of progression. The most frequently observed symptoms included blepharoedema, anorexia, tremors, dyspnoea, and ataxia characterized by circular movements or lateral instability (Table 1). Because of the severity and progression of these symptoms, the hedgehogs in group B were euthanized at 5 DPI, 15 DPI, 23 DPI, and 44 DPI.

Group A showed significant weight gain during the experiment (*p* = 2.89 × 10^−4^), while group B did not (*p* = 0.873), as confirmed by the Kruskal–Wallis test (χ^2^ = 9.26, *p* = 0.0097) (Table 1; Supporting Information S3).

### 3.2 *Angiostrongylus cantonensis* Infection Has Only Minimal Effects on Peripheral Blood Parameters

**FIGURE 2:**
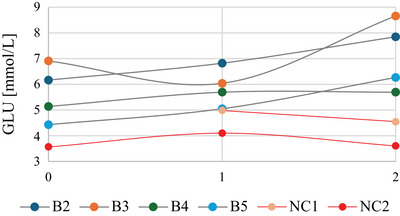
Graphical representation of the GLU values in the blood of experimentally infected hedgehogs (grey curves) compared to the negative controls (red curves). The missing value in the negative control (NC1) is due to a failed measurement of the biochemical analyzer. The values 0, 1, and 2 on the *x*‐axis correspond to the three measurement intervals (0 DPI, 15 DPI, and the day of euthanasia).

No major differences attributable to the infection were observed; only a significant increase in glucose (GLU) on the day of euthanasia in group B (χ^2^ = 7.23, *p* = 0.027, *p*.adj < 0.05) (Figure 2; Supporting Information S1). The rapid onset of clinical signs in hedgehog B1 prevented blood testing on the day of euthanasia, obstructing the assessment of the infection's effect on blood parameters.

### 3.3 Distribution of the DNA of *A. cantonensis* in Tissue of Hedgehogs

Quantitative PCR showed the presence of *A. cantonensis* DNA in various tissues of all infected hedgehogs (Table 2). The number of positive tissues increased over time in group A (*p* = 0.026), with no visible migration into the CNS including brain and CSF. In hedgehog B1 (euthanized on 5 DPI) almost all organs were positive. CSF could not be collected from this individual. In hedgehogs B2, B3, B4, and B5 (euthanized on the 15, 23, and 44 DPI), a decrease in the number of DNA‐positive tissues was observed, with positivity in the CNS. The decrease in the number of DNA‐positive tissues outside the CNS was statistically significant over time (*p* = 2.07 × 10^−7^). The estimated numbers of larvae in the tissue samples showed considerable variability, with the highest counts (Table 2) observed in the brain and CSF (B1–B5). The difference in larval counts between non‐CNS and CNS tissues was statistically significant (*p* = 0.038).

### 3.4 The Presence of *A. cantonensis* Larvae Is Associated With Inflammation in the CNS

**FIGURE 3:**
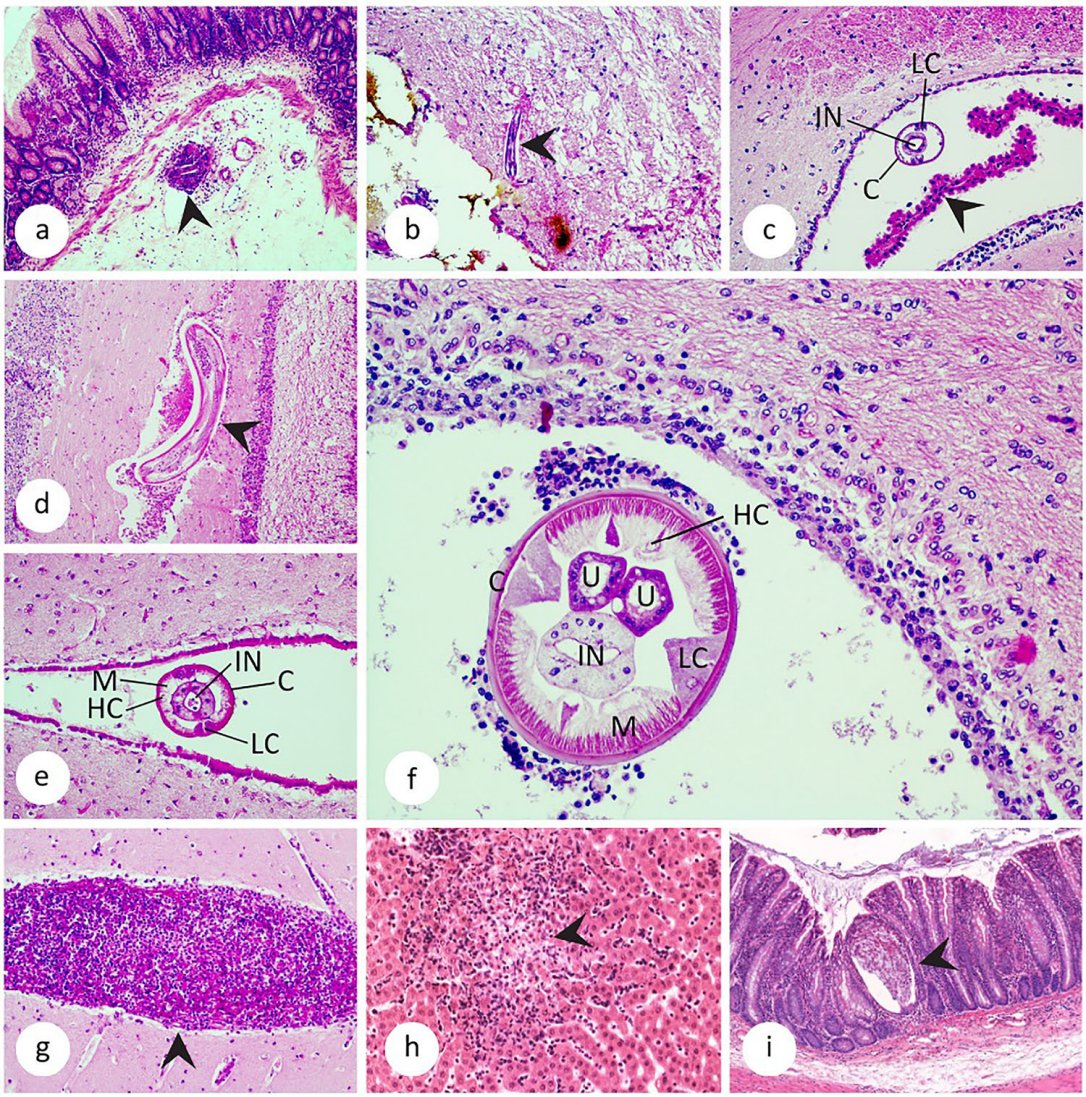
Sections of organs of experimentally infected hedgehogs inoculated with 200 and 2000 L3 of *A. cantonensis* (group A and B), stained with H&E, magnification: 100× (a, g, i), 200× (b, c, d, e, h), and 400× (f). (a): Longitudinal section of a larva (black arrow) in the lumen of the lymphatic vessel in the pyloric submucosa wrapped in inflammatory infiltrate comprising macrophages and small lymphocytes (B1, DPI 5, detail in Supporting Information S4), detailed; (b) longitudinal section of a larva (black arrow) in the brain white matter (B1, DPI 5); (c) cross‐section of a larva in the lateral brain chamber with choroid plexus marked by black arrow (B4, DPI 15); (d) longitudinal section of a larva (black arrow) in the molecular layer of the cerebellum surrounded by macrophages and small lymphocytes (B3, DPI 23); (e) cross‐section of a larva in the lateral brain chamber (B3, DPI 23); (f) cross‐section of a subadult or young adult stage of *A. cantonensis* in the lateral brain chamber (B5, DPI 44); (g) suppurative to pyogranulomatous meningitis, marked by black arrow (B3, DPI 23); (h) pyogranulomatous hepatitis, marked by black arrow (A4, DPI 50); (i) dilated rectal crypts (black arrow) with accumulation of necrotic material and cellular debris (crypt abcessi) (B4, DPI 15); morphological features: uterus (U), lateral cords (LC), intestine (IN), polymyarian musculature (M), accessory hypodermal cords (HC), and cuticle (C).

Histopathological examination revealed the presence of *A. cantonensis* larvae in group B hedgehogs, namely in the pylorus (B1, Figure 3a; Supporting Information S4), brain white matter (B1, Figure 3b), cerebellum (B1, Figure 3d), and brain chambers (B2–B5, Figure 3c,e,f); no nematodes were observed in group A tissue samples. The morphology of the larvae showed an intact cuticle, well‐developed polymyarian musculature, lateral chords, and gut, as well as gradual development of the reproductive organs and general larval growth over time in cross‐sections (Figure 3c,e,f). Gravid females were not observed in any of the hedgehogs.

The larvae in the tissues were often surrounded by macrophages and small lymphocytes (Figure 3c–g) including the larvae migrating via lymphatic vessels.

Histopathological analysis revealed additional abnormalities in the organ sections of hedgehog A4 and all hedgehogs of group B, such as pyogranulomatous hepatitis and rectal crypt abscesses (Figure 3h,i), liver lipidosis, and dilation of glomerular Bowman spaces in which no larvae were found (Table 1). The negative controls showed no pathological changes.

### 3.5 Absence of L1 in Hedgehog Feces

Coproscopic examination of feces did not reveal the presence of L1 in any of the experimental hedgehogs that survived to the 44 DPI (A2, A4, A6, and B5), which further confirms that hedgehogs are not permissive hosts of *A. cantonensis*. On the contrary, the excretion of L1 in feces was confirmed in the positive controls (two Wistar rats), which proves the infectivity of the L3 used.

## 4 Discussion

*A. cantonensis*, the causative agent of human neuroangiostrongyliasis of zoonotic origin, has gradually spread from Southeast Asia to the global tropics and subtropics (Cowie et al. 2023), with new populations thought to have recently been identified on the temperate Mediterranean island of Mallorca and mainland Spain (Jaume‐Ramis et al. 2023; Fuentes et al. 2024). The study experimentally builds on the fact that both reports of *A. cantonensis* in Mallorca described infection in free‐living Algerian hedgehogs *A. algirus*, making these free‐living mammals an excellent sentinel for the occurrence of *A. cantonensis* in this ecosystem (Delgado‐Serra et al. 2022). Our research consists of two consecutive experiments on a related species, the African pygmy hedgehog (*A. albiventris*), a commonly captive‐bred species, in which we studied the course of infection and the possible completion of the parasite's life cycle after inoculation with two different doses of *A. cantonensis* L3.

Hedgehogs inoculated with a low infectious dose (200 L3, group A) showed no clinical signs, gained weight over time, and thrived. In contrast, highly infected hedgehogs (2000 L3, group B) showed neurological disorders similar to those described for hedgehog infections in Mallorca (Delgado‐Serra et al. 2022; Paredes‐Esquivel et al. 2019) with histologically proven presence of *A. cantonensis* larvae in the brain, including cerebellum. The manifestation of clinical symptoms occurred gradually in all hedgehogs in this group, with varying progression and severity. The hedgehogs in group B did not thrive and showed only minimal weight gain or even weight loss. Similar neurological disorders associated with NA have been observed in aberrant hosts in the wild, for example, opossums (*Didelphis virginiana* Kerr, 1792), nine‐banded armadillos (*Dasypus novemcinctus* Linnaeus, 1758), and fruit bats (*Pteropus* spp.). In these cases, the symptoms were so severe that the animals were euthanized in rescue centers or died spontaneously within a few days of admission (Barret et al. 2002; Dalton et al. 2017).

Analysis of the peripheral blood revealed no major changes in the blood parameters in the experimental groups of hedgehogs. Only the analysis of GLU levels revealed elevated values in group B, which is consistent with observations in experimentally infected mice (Peng et al. 2024). This could indicate disturbances in glycolysis, an important metabolic pathway for energy production in the brain, due to the presence of *A. cantonensis* larvae in the brains of highly infected hedgehogs and damage to brain tissue. Infections with *A. cantonensis* in aberrant hosts are typically associated with eosinophilia (Gosnell and Kramer 2013; Lan et al. 2004; Martins et al. 2015); however, this phenomenon has not been documented in hedgehogs. This observation is consistent with horses and some human cases in which infection had not led to detectable changes in peripheral blood (Costa et al. 2000; Prociv and Turner 2018; Wright et al. 1991).

Histopathological examination revealed nematode larvae only in the tissues of the highly infected hedgehogs (group B), where the larvae were found in the subarachnoid space and parenchyma, which is consistent with previous findings in *A. algirus* in Mallorca (Arango‐Colonna et al. 2023). The larvae often incited granulomatous inflammation comprising mainly macrophages and lesser numbers of small lymphocytes, which is commonly described in tissue migrating nematode larvae (Dobey et al. 2014; Spratt 2015). Migration of larvae in the brain tissue most likely damaged the blood–brain barrier enabling development of suppurative to pyogranulomatous meningitis, most likely of a bacterial etiology. The presence of an intact cuticle and well‐preserved internal morphology of the larvae suggests that the larvae were viable at the time of examination. The development of the larvae can also be recognized by their size growth and the development of the reproductive system, although no gravid females were detected. The presence of larvae and character of the lesions were consistent with the dispersal and subsequent migration of larvae to the brain, as has been repeatedly described (Jarvi and Prociv 2021; Martins et al. 2015; Wang et al. 2015). In addition, pathological changes were also observed in other tissues (including low‐infected hedgehog A4). These changes may be attributed to the migration route of the larvae or to larvae that were lost during migration, died, and disintegrate (Jarvi and Prociv 2021; Prociv and Turner 2018).

The presence of *A. cantonensis* DNA was confirmed in tissues of both groups of hedgehogs. Low‐infected hedgehogs (group A) showed an increasing number of DNA‐positive tissues over time, without signs of neurotropism and without histopathologically detectable larvae. It is possible that the larvae were lysed, releasing a larger amount of DNA (Jarvi et al. 2015, 2024), which is reflected in the increased number of DNA‐positive tissues. A different pattern was observed in the highly infected hedgehogs (group B), in which the number of DNA‐positive tissues decreased over time, accompanied by the presence of larvae in the CNS at the end of the experiment. Quantitative analysis showed that the highest concentrations of larvae in group B were localized in the brain and CSF, while the amount of DNA in other tissues was very low or negligible, possibly because of the ability of larvae to release some DNA during migration (Jarvi et al. 2015). Design of our study cannot fully explain discrepancies between the DNA presence and absence of larvae in histological sections from the given organ and is probably related to generally low numbers of larvae. Higher sensitivity of the PCR is probably attributable to larger volume of tissue used for the DNA isolation and/or to the remnants of parasite fragment of DNA in tissues.

Neuroangiostrongyliasis in humans and other aberrant hosts is the result of a unique parasite migration. The L3 typically migrate from the intestine through the liver and lungs via the bloodstream and successfully reach the CNS shortly after infection (Cowie 2013; Pan et al. 2000; Wang et al. 2006). In rats, at least two thirds of the larvae complete migration through the CNS, indicating a very strong neurotropism. As a result, inocula with more than 80–100 larvae lead to clinical symptoms or even death in experimental rats (Ji et al. 2017; Kwong and Dobson 1982; Wallace and Rosen 1969; Wang et al. 2015). This is in stark contrast to the situation we described in hedgehogs, in which only a small proportion of the larvae reached the brain and continued to develop. The fate of the rest of the larvae remains unknown; they are probably excreted via the gastrointestinal tract or lysed in the blood during dispersal (Jarvi et al. 2015, 2024).

Previous studies of *A. algirus* showed the presence of fully developed *A. cantonensis* in the subarachnoid space, including an adult gravid female (Delgado‐Serra et al. 2022; Paredes‐Esquivel et al. 2019), which raises the question of a possible role of hedgehogs in the life cycle and dissemination of the parasite. However, the absence of adult worms in the circulatory system and the absence of L1 in the lungs and feces after the expected prepatent period in our experimental animals do not indicate that hedgehogs are an alternative to rats as a definitive host. Rather, hedgehogs act as aberrant dead‐end hosts, similar to other homoiotherms, including humans (Lunn et al. 2012; Spratt 2015; Walden et al. 2021; Wang et al. 2008). Our study does confirm previous reports on the maturation of *A. cantonensis* females in aberrant hosts (McKenzie et al. 1978; Paredes‐Esquivel et al. 2019; Patial et al. 2022), albeit without evidence of reproductive capacity. This is further confirmed by the absence of gravid females in a subsequent study in hedgehog on Mallorca (Arango‐Colonna et al. 2023).

In naturally infected hedgehogs and other wild mammals reported as aberrant hosts of *A. cantonensis*, the infection was identified based on clinical manifestations, although the mode of infection and its time course remained unknown (Barrett et al. 2002; Dalton et al. 2017; Delgado‐Serra et al. 2022; Kim et al. 2002). However, the clinical cases described in wild animals probably represent only a small fraction of the total number of infections, as low infection doses may not cause obvious clinical symptoms and therefore remain undetected, as shown by our results in the low‐dose hedgehogs. The geographical distribution and range of aberrant hosts identified also suggest socio‐cultural biases. Most cases in wild mammals and birds have been described in the United States and Australia, countries with developed wildlife disease surveillance systems and a public sensitized to wildlife conservation and One Health. In contrast, reports of *A. cantonensis* infections in wildlife in Asia are negligible, despite the widespread distribution of the parasite in this region and numerous clinical cases in humans (Cowie et al. 2022; Kodama et al. 1981; Spratt 2015; Walden et al. 2021).

Our experimental results suggest that free ranging hedgehogs showing neuropathological manifestations under natural conditions are only those exposed to high numbers of larvae from consumed natural food (gastropods, reptiles, amphibians, or crustaceans), and it is likely that a considerable number of infected animals survive. Based on the number of larvae found in the brains of Mallorca hedgehogs (up to 68 larvae per hedgehog), we conclude that wild hedgehogs probably ingested a much larger number of larvae, as their parasite load in the brain was significantly higher than the few larvae observed in experimental hedgehogs (Arango‐Colonna et al. 2023). Assessing the actual impact of infection on insectivore populations is problematic. However, it is likely that severe cases following occasional ingestion of high amounts of L3 in consumed intermediate or paratenic hosts may be observed in a range of vertebrate species in newly established infection foci.

Notable clinical manifestations in hedgehogs have enabled the discovery and subsequent surveillance of a new *A. cantonensis* focus on Mallorca, supported by collaboration among zoologists, parasitologists, wildlife rescue centers, and public (Delgado‐Serra et al. 2022; Paredes‐Esquivel et al. 2019). Based on new data obtained, hedgehogs are not permissive hosts of *A. cantonensis* and do not support its circulation in the Balearic terrestrial ecosystem. However, targeted surveillance of neurological disorders in wildlife, including hedgehogs, may provide further information on the distribution of *A. cantonensis* in Mediterranean Europe, its possible future spread to temperate latitudes and risk of emergence of the clinical disease in human population.

## Conflicts of Interest

The authors declare no conflicts of interest.

## Supporting information




**Supporting Information S1**: Results of the biochemical blood tests of the two experimental groups (A and B) and the negative control group; measured parameters: Glucose (GLU), creatinine (CREA), albumin (ALB), globulin (GLOB), alanine transaminase (ALT), aspartate transferase (AST), alkaline phosphatase (ALP)

**Supporting Information S2**: Results of the hematological blood tests of the two experimental groups (A and B) and the negative control group; measured parameters: Red blood cells (RBC), hemoglobin (HGB), mean corpuscular volume (MCV), mean corpuscular hemoglobin (MCH), mean corpuscular hemoglobin concentration (MCHC), white blood cells (WBC), percentage of neutrophils (%NEU), percentage of lymphocytes (%LYM), percentage of monocytes (%MONO), percentage of eosinophils (%EOS), percentage of basophils (%BASO), neutrophil count (NEU), lymphocyte count (LYM), monocyte count (MONO), eosinophil count (EOS), basophil count (BASO)

**Supporting Information S3**: Results of the weight measurements of the two experimental groups (A and B) and the negative control group

**Supporting Information S4**: Longitudinal‐section of the larva in the lumen of the lymphatic vessel in the pyloric submucosa wrapped in inflammatory infiltrate comprising macrophages and small lymphocytes (B1, DPI 5, magnification 200x)

## Data Availability

The data supporting the findings of this study are provided in the main text and supplementary materials.
