# Supplementary material for: Hedgehogs and Angiostrongylus cantonensis: Uncovering the Role of Atelerix albiventris in the Parasite Life Cycle
Source: Integr Zool. 2025 May 21;21(1):104–15. doi: 10.1111/1749-4877.13004 (PMC12794780; doi:10.1111/1749-4877.13004)
Supplement: Supplementary file 1 — Supporting Information S1: Results of the biochemical blood tests of the two experimental groups (A and B) and the negative control group; measured parameters: Glucose (GLU), creatinine (CREA), albumin (ALB), globulin (GLOB), alanine transaminase (ALT), aspartate transferase (AST), alkaline phosphatase (ALP) [file INZ2-21-104-s002.pdf]

Biochemical parameters

A group

A1 (male)

Parameter	0 DPI	15 DPI	30 DPI	50 DPI	Unit
**GLU**	4.94	6.93	3.67	NA	mmol/L
**CREA**	9	< 9	37	NA	µmol/L
**ALB**	36	36	40	NA	g/L
**GLOB**	30	33	32	NA	g/L
**ALT**	70	149	73	NA	U/L
**AST**	44	176	45	NA	U/L
**ALP**	72	62	72	NA	U/L

A2 (male)

Parameter	0 DPI	15 DPI	30 DPI	50 DPI	Unit
**GLU**	3.65	3.69	2.81	3.02	mmol/L
**CREA**	< 9	< 9	11	11	µmol/L
**ALB**	32	29	35	36	g/L
**GLOB**	28	29	30	25	g/L
**ALT**	56	72	68	37	U/L
**AST**	46	43	71	29	U/L
**ALP**	59	57	58	74	U/L

A3 (male)

Parameter	0 DPI	15 DPI	30 DPI	50 DPI	Unit
**GLU**	NA	5.2	5.61	NA	mmol/L
**CREA**	NA	10	< 9	NA	µmol/L
**ALB**	36	33	47	NA	g/L
**GLOB**	NA	34	46	NA	g/L
**ALT**	NA	234	126	NA	U/L
**AST**	77	82	157	NA	U/L
**ALP**	NA	99	11	NA	U/L

A4 (male)

Parameter	0 DPI	15 DPI	30 DPI	50 DPI	Unit
**GLU**	3.47	4.48	NA	5.71	mmol/L
**CREA**	< 9	< 9	NA	< 9	µmol/L
**ALB**	37	36	NA	40	g/L
**GLOB**	34	31	NA	35	g/L
**ALT**	72	90	NA	66	U/L
**AST**	41	131	NA	110	U/L
**ALP**	68	24	NA	57	U/L

A5 (female)

Parameter	0 DPI	15 DPI	30 DPI	50 DPI	Unit
**GLU**	3.56	4.09	3.6	NA	mmol/L
**CREA**	< 9	< 9	10	NA	µmol/L
**ALB**	33	34	41	NA	g/L
**GLOB**	29	28	30	NA	g/L
**ALT**	144	113	119	NA	U/L
**AST**	64	84	70	NA	U/L
**ALP**	29	61	49	NA	U/L

A6 (female)

Parameter	0 DPI	15 DPI	30 DPI	50 DPI	Unit
**GLU**	4.1	4.19	3.22	4.79	mmol/L
**CREA**	< 9	< 9	13	32	µmol/L
**ALB**	37	36	41	41	g/L
**GLOB**	32	32	31	26	g/L
**ALT**	511	189	178	63	U/L
**AST**	250	94	110	49	U/L
**ALP**	54	73	56	49	U/L

B group

B1 (male)

Parameter	0 DPI	15 DPI	23 DPI	44 DPI	Unit
**GLU**	2.69	NA	NA	NA	mmol/L
**CREA**	11	NA	NA	NA	µmol/L
**ALB**	31	NA	NA	NA	g/L
**GLOB**	29	NA	NA	NA	g/L
**ALT**	181	NA	NA	NA	U/L
**AST**	126	NA	NA	NA	U/L
**ALP**	84	NA	NA	NA	U/L

B2 (male)

Parameter	0 DPI	15 DPI	23 DPI	50 DPI	Unit
**GLU**	6.16	6.82	7.85	NA	mmol/L
**CREA**	< 9	< 9	< 9	NA	µmol/L
**ALB**	31	31	34	NA	g/L
**GLOB**	29	28	32	NA	g/L
**ALT**	41	46	40	NA	U/L
**AST**	41	34	58	NA	U/L
**ALP**	44	47	51	NA	U/L

B3 (male)

Parameter	0 DPI	15 DPI	23 DPI	50 DPI	Unit
**GLU**	6.91	6.04	8.66	NA	mmol/L
**CREA**	< 9	11	< 9	NA	µmol/L
**ALB**	30	31	36	NA	g/L
**GLOB**	35	38	41	NA	g/L
**ALT**	101	107	61	NA	U/L
**AST**	56	74	80	NA	U/L
**ALP**	48	45	54	NA	U/L

B4 (male)

Parameter	0 DPI	15 DPI	23 DPI	50 DPI	Unit
**GLU**	5.14	5.69	NA	NA	mmol/L
**CREA**	< 9	13	NA	NA	µmol/L
**ALB**	33	33	NA	NA	g/L
**GLOB**	37	37	NA	NA	g/L
**ALT**	83	60	NA	NA	U/L
**AST**	104	86	NA	NA	U/L
**ALP**	45	57	NA	NA	U/L

B5 (male)

Parameter	0 DPI	15 DPI	44 DPI	50 DPI	Unit
**GLU**	4.42	5.04	6.26	NA	mmol/L
**CREA**	< 9	13	13	NA	µmol/L
**ALB**	35	37	33	NA	g/L
**GLOB**	35	35	36	NA	g/L
**ALT**	66	55	40	NA	U/L
**AST**	84	55	50	NA	U/L
**ALP**	10	41	41	NA	U/L

Negative control group

NC1 (female)

Parameter	0 DPI	15 DPI	30 DPI	50 DPI	Unit
**GLU**	3.93	4.81	4.2	NA	mmol/L
**CREA**	11	17	36	NA	µmol/L
**ALB**	34	28	37	NA	g/L
**GLOB**	29	28	27	NA	g/L
**ALT**	76	77	100	NA	U/L
**AST**	80	107	100	NA	U/L
**ALP**	57	40	53	NA	U/L

NC2 (female)

Parameter	0 DPI	15 DPI	30 DPI	50 DPI	Unit
**GLU**	NA	4.98	4.54	NA	mmol/L
**CREA**	NA	<9	11	NA	µmol/L
**ALB**	NA	39	44	NA	g/L
**GLOB**	NA	35	31	NA	g/L
**ALT**	NA	74	93	NA	U/L
**AST**	NA	149	58	NA	U/L
**ALP**	NA	56	62	NA	U/L

Supporting information 1 Results of the biochemical blood tests of the two experimental groups (A and B) and the negative control group; measured parameters: Glucose (GLU), creatinine (CREA), albumin (ALB), globulin (GLOB), alanine transaminase (ALT), aspartate transferase (AST), alkaline phosphatase (ALP)
