# Supplementary material for: Hedgehogs and Angiostrongylus cantonensis: Uncovering the Role of Atelerix albiventris in the Parasite Life Cycle
Source: Integr Zool. 2025 May 21;21(1):104–15. doi: 10.1111/1749-4877.13004 (PMC12794780; doi:10.1111/1749-4877.13004)
Supplement: Supplementary file 2 — Supporting Information S2: Results of the hematological blood tests of the two experimental groups (A and B) and the negative control group; measured parameters: Red blood cells (RBC), hemoglobin (HGB), mean corpuscular volume (MCV), mean corpuscular hemoglobin (MCH), mean corpuscular hemoglobin concentration (MCHC), white blood cells (WBC), percentage of neutrophils (%NEU), percentage of lymphocytes (%LYM), percentage of monocytes (%MONO), percentage of eosinophils (%EOS), percentage of basophils (%BASO), neutrophil count (NEU), lymphocyte count (LYM), monocyte count (MONO), eosinophil count (EOS), basophil count (BASO) [file INZ2-21-104-s001.pdf]

Haematological parameters

A group

A1 (male)

Parameter	0 DPI	15 DPI	30 DPI	50 DPI	Units
**RBC**	6.24	3.84	5.52	NA	x10^12/L
**HGB**	13.3	8.5	11.9	NA	g/dL
**MCV**	67	67.4	68.3	NA	fL
**MCH**	21.3	22.1	21.6	NA	pg
**MCHC**	31.8	32.8	31.6	NA	g/dL
**WBC**	5.21	3.01	5.46	NA	x10^9/L
**%NEU**	1.5	1.7	1.8	NA	%
**%LYM**	86.4	86.7	84.8	NA	%
**%MONO**	9.2	8.3	9.7	NA	%
**%EOS**	2.5	3.3	3.5	NA	%
**%BASO**	0.4	0	0.2	NA	%
**NEU**	0.08	0.05	0.1	NA	x10^9/L
**LYM**	4.5	2.61	4.63	NA	x10^9/L
**MONO**	0.48	0.25	0.53	NA	x10^9/L
**EOS**	0.13	0.1	0.19	NA	x10^9/L
**BASO**	0.02	0	0.01	NA	x10^9/L

A2 (male)

Parameter	0 DPI	15 DPI	30 DPI	50 DPI	Units
**RBC**	12.43	4.39	5.32	6.44	x10^12/L
**HGB**	27.9	10.1	12.5	14.8	g/dL
**MCV**	75.3	74.3	78.4	77.8	fL
**MCH**	22.4	23	23.5	23	pg
**MCHC**	29.8	31	30	29.5	g/dL
**WBC**	3.66	11.34	11.11	11.23	x10^9/L
**%NEU**	1.1	0.6	1.5	0.7	%
**%LYM**	86.3	87.6	86.2	85.2	%
**%MONO**	9.3	9	9.4	12.6	%
**%EOS**	3	2.6	2.6	1.3	%
**%BASO**	0.04	0.2	0.3	0.2	%
**NEU**	0.04	0.08	0.17	0.07	x10^9/L
**LYM**	3.16	9.93	9.58	9.57	x10^9/L
**MONO**	0.34	1.02	1.04	1.42	x10^9/L
**EOS**	0.11	0.29	0.29	0.15	x10^9/L
**BASO**	0.01	0.02	0.03	0.02	x10^9/L

A3 (male)

Parameter	0 DPI	15 DPI	30 DPI	50 DPI	Units
**RBC**	11.84	4.21	2.41	NA	x10^12/L
**HGB**	26.4	9.4	5.5	NA	g/dL
**MCV**	77.1	73.9	71.4	NA	fL
**MCH**	22.3	22.3	22.8	NA	pg
**MCHC**	28.9	30.2	32	NA	g/dL
**WBC**	1.68	6.08	1.41	NA	x10^9/L
**%NEU**	1.1	39.7	61	NA	%
**%LYM**	76.2	46.7	31.9	NA	%
**%MONO**	17.9	9.9	5.7	NA	%
**%EOS**	3.6	2.1	1.4	NA	%
**%BASO**	1.2	1.6	0	NA	%
**NEU**	0.02	2.41	0.86	NA	x10^9/L
**LYM**	1.28	2.84	0.45	NA	x10^9/L
**MONO**	0.3	0.6	0.08	NA	x10^9/L
**EOS**	0.06	0.13	0.02	NA	x10^9/L
**BASO**	0.02	0.1	0	NA	x10^9/L

A4 (male)

Parameter	0 DPI	15 DPI	30 DPI	50 DPI	Units
**RBC**	7.51	4.13	0.1	6.82	x10^12/L
**HGB**	15.9	8.9	0.8	14.6	g/dL
**MCV**	69.2	70.7	50	69.6	fL
**MCH**	21.2	21.5	80	21.4	pg
**MCHC**	30.6	30.5	160	30.7	g/dL
**WBC**	5.18	5.67	0.01	12.35	x10^9/L
**%NEU**	52.8	40.2	100	52	%
**%LYM**	33.8	47.6	0	37.1	%
**%MONO**	9.5	8.5	0	10	%
**%EOS**	2.5	2.5	0	0.6	%
**%BASO**	1.4	1.2	0	0.3	%
**NEU**	2.74	2.28	0.01	6.42	x10^9/L
**LYM**	1.75	2.7	0	4.58	x10^9/L
**MONO**	0.49	0.48	0	1.23	x10^9/L
**EOS**	0.13	0.14	0	0.08	x10^9/L
**BASO**	0.07	0.07	0	0.04	x10^9/L

B group

B1 (male)

Parameter	0 DPI	15 DPI	23 DPI	44 DPI	Units
**RBC**	5.82	NA	NA	NA	x10^12/L
**HGB**	11.7	NA	NA	NA	g/dL
**MCV**	62.5	NA	NA	NA	fL
**MCH**	20.1	NA	NA	NA	pg
**MCHC**	32.1	NA	NA	NA	g/dL
**WBC**	9.68	NA	NA	NA	x10^9/L
**%NEU**	44.9	NA	NA	NA	%
**%LYM**	46.9	NA	NA	NA	%
**%MONO**	5	NA	NA	NA	%
**%EOS**	3.1	NA	NA	NA	%
**%BASO**	0.1	NA	NA	NA	%
**NEU**	4.35	NA	NA	NA	x10^9/L
**LYM**	4.54	NA	NA	NA	x10^9/L
**MONO**	0.48	NA	NA	NA	x10^9/L
**EOS**	0.3	NA	NA	NA	x10^9/L
**BASO**	0.01	NA	NA	NA	x10^9/L

B2 (male)

Parameter	0 DPI	15 DPI	23 DPI	44 DPI	Units
**RBC**	6.2	5.62	7.91	NA	x10^12/L
**HGB**	12.1	11.3	16.2	NA	g/dL
**MCV**	61.8	64.4	68.5	NA	fL
**MCH**	19.5	20.1	20.5	NA	pg
**MCHC**	31.6	31.2	29.9	NA	g/dL
**WBC**	6.34	8.61	5.13	NA	x10^9/L
**%NEU**	52.2	43	56.9	NA	%
**%LYM**	33.1	43.2	31	NA	%
**%MONO**	13.1	11.5	10.3	NA	%
**%EOS**	1.1	1.5	1.2	NA	%
**%BASO**	0.5	0.8	0.6	NA	%
**NEU**	3.31	3.7	2.92	NA	x10^9/L
**LYM**	2.1	3.72	1.59	NA	x10^9/L
**MONO**	0.83	0.99	0.53	NA	x10^9/L
**EOS**	0.07	0.13	0.06	NA	x10^9/L
**BASO**	0.03	0.07	0.03	NA	x10^9/L

B3 (male)

Parameter	0 DPI	15 DPI	23 DPI	44 DPI	Units
**RBC**	5.91	6.28	7.87	NA	x10^12/L
**HGB**	11.8	12.5	15.9	NA	g/dL
**MCV**	63.5	65	64.7	NA	fL
**MCH**	20	19.9	20.2	NA	pg
**MCHC**	31.5	30.6	31.2	NA	g/dL
**WBC**	4.66	3.66	2.61	NA	x10^9/L
**%NEU**	49.2	2.6	3	NA	%
**%LYM**	36.7	68.6	72.8	NA	%
**%MONO**	12.2	23	19.2	NA	%
**%EOS**	1.5	5.5	4.6	NA	%
**%BASO**	0.4	0.3	0.4	NA	%
**NEU**	2.29	0.1	0.08	NA	x10^9/L
**LYM**	1.71	2.51	1.9	NA	x10^9/L
**MONO**	0.57	0.84	0.5	NA	x10^9/L
**EOS**	0.07	0.2	0.12	NA	x10^9/L
**BASO**	0.02	0.01	0.01	NA	x10^9/L

B4 (male)

Parameter	0 DPI	15 DPI	23 DPI	44 DPI	Units
**RBC**	6.83	5.78	NA	NA	x10^12/L
**HGB**	13.7	11.7	NA	NA	g/dL
**MCV**	66.8	68.7	NA	NA	fL
**MCH**	20.1	20.2	NA	NA	pg
**MCHC**	30	29.5	NA	NA	g/dL
**WBC**	8.95	10.8	NA	NA	x10^9/L
**%NEU**	3.6	49.3	NA	NA	%
**%LYM**	70.5	36.5	NA	NA	%
**%MONO**	20.8	11.6	NA	NA	%
**%EOS**	4.9	2.4	NA	NA	%
**%BASO**	0.2	0.2	NA	NA	%
**NEU**	0.32	5.33	NA	NA	x10^9/L
**LYM**	6.31	3.94	NA	NA	x10^9/L
**MONO**	1.86	1.25	NA	NA	x10^9/L
**EOS**	0.44	0.26	NA	NA	x10^9/L
**BASO**	0.02	0.02	NA	NA	x10^9/L

A5 (female)					
**Parameter**	**0 DPI**	**15 DPI**	**30 DPI**	**50 DPI**	**Units**
**RBC**	4.52	3.56	4.42	NA	x10^12/L
**HGB**	11.2	8.6	10.9	NA	g/dL
**MCV**	84.3	81.7	83.7	NA	fL
**MCH**	24.8	24.2	24.7	NA	pg
**MCHC**	29.4	29.6	29.5	NA	g/dL
**WBC**	8.65	5.57	3.8	NA	x10^9/L
**%NEU**	64.1	47.4	46.3	NA	%
**%LYM**	28.2	42.5	44.5	NA	%
**%MONO**	6.4	8.1	6.1	NA	%
**%EOS**	0.8	1.6	1.3	NA	%
**%BASO**	0.5	0.4	1.8	NA	%
**NEU**	5.55	2.64	1.76	NA	x10^9/L
**LYM**	2.44	2.37	1.69	NA	x10^9/L
**MONO**	0.55	0.45	0.23	NA	x10^9/L
**EOS**	0.07	0.09	0.05	NA	x10^9/L
**BASO**	0.04	0.02	0.07	NA	x10^9/L

B5 (male)					
**Parameter**	**0 DPI**	**15 DPI**	**23 DPI**	**44 DPI**	**Units**
**RBC**	6.17	6.54	6.8	NA	x10^12/L
**HGB**	10.8	11.5	12.2	NA	g/dL
**MCV**	55.4	56	55.6	NA	fL
**MCH**	17.5	17.6	17.9	NA	pg
**MCHC**	31.6	31.4	32.3	NA	g/dL
**WBC**	7.93	6.29	8.23	NA	x10^9/L
**%NEU**	4.9	3	2.9	NA	%
**%LYM**	75.3	77.3	65	NA	%
**%MONO**	15.8	15.4	19.1	NA	%
**%EOS**	3.9	4.1	12.9	NA	%
**%BASO**	0.1	0.2	0.1	NA	%
**NEU**	0.39	0.19	0.24	NA	x10^9/L
**LYM**	5.97	4.86	5.35	NA	x10^9/L
**MONO**	1.25	0.97	1.57	NA	x10^9/L
**EOS**	0.31	0.26	1.06	NA	x10^9/L
**BASO**	0.01	0.01	0.01	NA	x10^9/L

A6 (female)					
**Parameter**	**0 DPI**	**15 DPI**	**30 DPI**	**50 DPI**	**Units**
**RBC**	8.66	5.19	4.59	5.66	x10^12/L
**HGB**	21.2	12.4	10.8	13	g/dL
**MCV**	81.9	78.4	76	73.9	fL
**MCH**	24.5	23.9	23.5	23	pg
**MCHC**	29.9	30.5	30.9	31.1	g/dL
**WBC**	1.42	6.47	6.53	4.21	x10^9/L
**%NEU**	3.5	38.5	65.2	2.7	%
**%LYM**	66.2	48.8	25.7	64.8	%
**%MONO**	27.5	11.6	8.1	31.1	%
**%EOS**	2.1	0.8	0.5	1.2	%
**%BASO**	0.7	0.3	0.5	0.2	%
**NEU**	0.05	2.49	4.26	0.11	x10^9/L
**LYM**	0.94	3.16	1.68	2.73	x10^9/L
**MONO**	0.39	0.75	0.53	1.31	x10^9/L
**EOS**	0.03	0.05	0.03	0.05	x10^9/L
**BASO**	0.01	0.02	0.03	0.01	x10^9/L

Negative control group

NC1 (female)					
**Parameter**	**0 DPI**	**15 DPI**	**30 DPI**	**50 DPI**	**Units**
**RBC**	5.32	5.54	0.05	NA	x10^12/L
**HGB**	11.2	11.6	0	NA	g/dL
**MCV**	67.5	69.1	60	NA	fL
**MCH**	21.1	20.9	0	NA	pg
**MCHC**	31.2	30.3	0	NA	g/dL
**WBC**	10.52	13.05	0.08	NA	x10^9/L
**%NEU**	56.5	50	NA	NA	%
**%LYM**	34.8	39.8	NA	NA	%
**%MONO**	5.9	7	NA	NA	%
**%EOS**	1.2	1.7	NA	NA	%
**%BASO**	1.6	1.5	NA	NA	%
**NEU**	5.94	6.53	NA	NA	x10^9/L
**LYM**	3.66	5.19	NA	NA	x10^9/L
**MONO**	0.62	0.91	NA	NA	x10^9/L
**EOS**	0.13	0.22	NA	NA	x10^9/L
**BASO**	0.17	0.2	NA	NA	x10^9/L

NC2 (female)					
**Parameter**	**0 DPI**	**15 DPI**	**30 DPI**	**50 DPI**	**Units**
**RBC**	4.97	1.8	4.4	NA	x10^12/L
**HGB**	12	4.3	10.5	NA	g/dL
**MCV**	82.7	86.1	86.1	NA	fL
**MCH**	24.1	23.9	23.9	NA	pg
**MCHC**	29.2	27.7	27.7	NA	g/dL
**WBC**	5.92	2.89	4.72	NA	x10^9/L
**%NEU**	47.7	61	1.7	NA	%
**%LYM**	40	30.4	78.8	NA	%
**%MONO**	9.1	6.6	14.4	NA	%
**%EOS**	1.7	1.7	3.6	NA	%
**%BASO**	1.5	0.3	1.5	NA	%
**NEU**	2.82	1.76	0.08	NA	x10^9/L
**LYM**	2.37	0.88	3.72	NA	x10^9/L
**MONO**	0.54	0.19	0.68	NA	x10^9/L
**EOS**	0.1	0.05	0.17	NA	x10^9/L
**BASO**	0.09	0.01	0.07	NA	x10^9/L

Supporting information 2 Results of the haematological blood tests of the two experimental groups (A and B) and the negative control group; measured parameters: Red blood cells (RBC), haemoglobin (HGB), mean corpuscular volume (MCV), mean corpuscular haemoglobin (MCH), mean corpuscular haemoglobin concentration (MCHC), white blood cells (WBC), percentage of neutrophils (%NEU), percentage of lymphocytes (%LYM), percentage of monocytes (%MONO), percentage of eosinophils (%EOS), percentage of basophils (%BASO), neutrophil count (NEU), lymphocyte count (LYM), monocyte count (MONO), eosinophil count (EOS), basophil count (BASO)
