# Supplementary material for: Hedgehogs and Angiostrongylus cantonensis: Uncovering the Role of Atelerix albiventris in the Parasite Life Cycle
Source: Integr Zool. 2025 May 21;21(1):104–15. doi: 10.1111/1749-4877.13004 (PMC12794780; doi:10.1111/1749-4877.13004)
Supplement: Supplementary file 3 — Supporting Information S3: Results of the weight measurements of the two experimental groups (A and B) and the negative control group [file INZ2-21-104-s004.pdf]

Hedgehog	Weight at 0 DPI [g]	Weight at 15 DPI [g]	Last weight [g]	Weight difference [g]
**A1***	434	478	478	44
**A2***	369	405	405	36
**A3***	379	424	424	45
**A4***	342	386	386	44
**A5***	353	396	396	43
**A6***	362	380	380	18
**B1**	295	NA	NA	NA
**B2**	333	343	343	10
**B3**	330	322	322	-8
**B4**	415	421	421	6
**B5**	441	436	436	-5
**NC1**	347	404	404	57
**NC2**	532	587	587	55

Supporting information 3 Results of the weight measurements of the two experimental groups (A and B) and the negative control group. The "weight at 0 DPI" corresponds to the measurement on the day of infection, and the "last weight" corresponds to the measurement on the day of euthanasia. The weight difference represents the difference between the "weight at 0 DPI" and the "last weight"; an asterisk indicates statistically significant weight changes *: $P < 0.05$
