# Supplementary figures and images for: Hedgehogs and Angiostrongylus cantonensis: Uncovering the Role of Atelerix albiventris in the Parasite Life Cycle
Source: Integr Zool. 2025 May 21;21(1):104–15. doi: 10.1111/1749-4877.13004 (PMC12794780; doi:10.1111/1749-4877.13004)

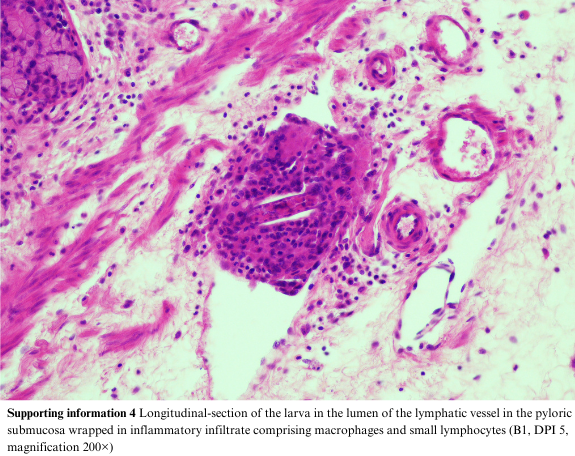

Supplement: Supplementary file 4 — Supporting Information S4: Longitudinal‐section of the larva in the lumen of the lymphatic vessel in the pyloric submucosa wrapped in inflammatory infiltrate comprising macrophages and small lymphocytes (B1, DPI 5, magnification 200x) [file INZ2-21-104-s003.tiff]
